# Direct measurement of two-photon absorption and refraction properties of SZ2080^TM^-based resists at 515 nm: insights into 3D printing

**DOI:** 10.1515/nanoph-2025-0066

**Published:** 2025-08-05

**Authors:** Michalis Stavrou, Dimitra Ladika, Edvinas Skliutas, Vytautas Jukna, David Gray, Maria Farsari, Saulius Juodkazis, Mangirdas Malinauskas

**Affiliations:** Foundation for Research and Technology-Hellas, Institute of Electronic Structure and Laser, Heraklion 70013, Greece; Laser Nanophotonics Group, Laser Research Center, Physics Faculty, Vilnius University, Vilnius, Lithuania; Optical Sciences Centre and ARC Training Centre in Surface Engineering for Advanced Materials (SEAM), School of Science, Swinburne University of Technology, Melbourne, Australia; WRH Program International Research Frontiers Initiative (IRFI) Tokyo Institute of Technology, Nagatsuta-cho, Midori-ku, Yokohama, Japan

**Keywords:** optical characterization, non-linear optics, laser-matter interaction, 3D printing, additive manufacturing, microstructures

## Abstract

Accurate knowledge of nonlinear optical parameters is essential for optimizing energy deposition in ultrafast laser 3D printing, yet these values remain undetermined for many commonly used materials. In this study, we address this gap by experimentally determining the two-photon absorption (TPA) and non-linear refraction coefficients (*β* and *n*_2_) of the widely used SZ2080^TM^ resist with the photo-initiators (PI) IRG369 and BIS (Irgacure 369 and 4,4′ bis(diethylamino)-benzophenone or Michler’s ketone). Using the Z-scan method at 515 nm with a low repetition rate (1 kHz) to avoid thermal accumulation, we found that the nonlinear response of the host polymer has a considerable contribution to energy deposition despite the addition of the PI, as the host polymer makes up the majority of 99 % in the solution. The TPA cross section *σ* were 5.7 ± 0.4 GM (1 GM = 10^−50^ cm^4^ s photon^−1^) for pure SZ2080^TM^, ∼40 GM for IRG and ∼87 GM for BIS at 515 nm. The nonlinear refractive index *n*_2_ for pure polymer was (85.3 ± 6) × 10^−5^ cm^2^/TW, favoring a self-focusing, and was larger than that for PIs: $∼16\times 10^{-5}$ cm^2^/TW (IRG369) and $∼2.8\times 10^{-5}$ cm^2^/TW (BIS). Hence, the properties of the host material govern non-linear light propagation, although, in high numerical aperture focusing, self-focusing has a minor contribution to the variation of refractive index. Crucially, the determined TPA coefficients for pure SZ2080^TM^ provide experimental evidence that it can initiate polymerization without PIs, enabling a more sustainable and environmentally friendly fabrication route by avoiding the use of toxic additive compounds. These findings will allow for the estimation of exact energy deposition in 3D laser printing using ultrashort laser pulses and support the development of an initiator-free additive manufacturing approach.

## 1 Introduction

3D printing (additive manufacturing) is undoubtedly a modern trend (from 2000) in industrial applications, originating from non-industrial prototyping and proof-of-the-concept approaches [1], [2], [3], [4]. The maturity of the technology streamlines the transition from laboratory trials to fabrication, which previously required much longer development and testing periods [5]. The present 3D printing technology conforms with the near-net-shaped 3D manufacturing approach, where minimal and simple post-processing steps are required to produce the final result/product. This also complies with sustainability and waste reduction trends [6].

For 3D printing driven by linear absorption of light, the absorption depth (or skin depth) *l*_*s*_ = 1/*α* defines the axial extent of solidified volume (in the light propagation direction), while the lateral extent is defined by the spatial distribution of the light in a projection or direct write of 3D printing; here *α* is the absorption coefficient. When ultra-short sub-1 ps pulses are used for 3D printing, the nonlinear (intensity *I* dependent) absorption and energy deposition can be utilized as the focused beam in the material can reach high intensities inside the material and initiates 3D printing *in-situ* [7], [8]. In this case, the nonlinear energy deposition will define the solidification of the material.

From a material property point of view, the absorption coefficient is a function dependent on intensity. The two-photon absorption (TPA) is accounted for by the expansion of the absorption as *α* = *α*_0_ + *βI*, where *α*_0_ is the linear absorption coefficient (intensity independent), and *β* is TPA coefficient [9], [10]. To aid the curation of a polymer, a small amount of the photo-initiator (PI) is added, typically at a 1 % vol (or wt) ratio. This addition improves the deposition of light energy because they have an absorption where the polymer is transparent. This translates to either reducing the order of the multiphoton absorption or, plainly, the material starts to be opaque to the radiation and absorbs the light linearly. It is especially noticeable for direct 3D polymerization by a lamp or LED exposure [11], [12], [13], [14], where the PI concentration is used to alter the linear absorption coefficient.

When ultra-short laser pulses are used for 3D polymerization, the PI can act as a seed for polymerization by altering the range of probable nonlinear responses, which also affect the host material. Indeed, the multi-photon absorption (MPA), impact (avalanche or inverse bremsstrahlung), and avalanche absorption seeded by PI (a stronger absorption) can lead to different radical formation pathways including ionization of the pre-polymer matrix. Although the fraction of PI in the solution is only *f* ≈ 1 % vol, the deposition of the laser energy, and consequently, the polymerized volume can drastically change. Mixing the PI in the host polymer reduces the absorption depth; therefore, lower pulse intensities are needed to reach the same energy deposition density. The concomitant nonlinear material effect observed at high intensities is the alteration of the refractive index of the material by the light itself. This effect, referred to as the Kerr effect, alters the material refractive index nonlinearly, expressed as *n* = *n*_0_ + *n*_2_ *I*, where *n*_0_ is the linear refractive index and *n*_2_ is the nonlinear refractive index. The change of the refractive index can alter the reflectivity of the material and can also cause self-focusing when *n*_2_ > 0 or self-defocusing when *n*_2_ < 0 [15]. These nonlinear effects (MPA and Kerr) are very fast material responses because they are driven by instantaneous electronic responses. The MPA and nonlinear refractive index coefficient alterations with the PI influence energy deposition in the focal volume of the incident laser pulse. This was experimentally validated in 3D printing of non-photosensitized and photosensitized resins using both amplified-pulse lasers [16] and high-repetition-rate ultrafast laser oscillators [17].

The most popular technique of nonlinear polymerization is through the use of the TPA, as it needs the lowest amount of intensity to initiate the nonlinear photo-curation, and it defines the precision in lateral and axial extend of photo-physical/chemical modifications [3], [18], [19], [20], [21], [22]. However, a major limitation in advancing high-resolution and energy-efficient 3D laser printing is the lack of experimentally determined nonlinear coefficients *β* and *n*_2_ for pure and photosensitized resist. These parameters are usually overlooked in the community due the lack of standardized methods for their measurement, prompting a reliance on empirical parametric approaches instead. Consequently, it becomes difficult to accurately understand how laser energy is absorbed and distributed within the material, or to determine whether interactions between the resist and photoinitiator influence energy deposition, making it challenging to optimize printing conditions in a reliable, predictive way. To address this challenge and accelerate process optimization, the nonlinear parameters have to be determined, which was a motivation for our research. Therefore, we have chosen to focus on the widely available and frequently used SZ2080^TM^ pre-polymer and IRG369 and BIS photosensitizers with typical conditions of laser 3D printing in terms of wavelength, pulse duration, and intensity. The nonlinear properties of the pure host SZ2080^TM^ pre-polymer were also investigated as there is an expected trend to reduce the use of UV photoinitiators in the leading industries of 3D printing in the EU [23].

In this study, we have retrieved the nonlinear optical responses (*β* and *n*_2_ coefficients) of the SZ2080^TM^ pre-polymer with and without the photoinitiators using the Z-scan technique. In our experiments we reached intensities of up to 1.8 TW/cm^2^, corresponding to the maximum usable intensity for pure SZ2080^TM^, beyond which nonlinear ionization and material breakdown occur. The onset of polymerization for pure SZ2080^TM^ was experimentally observed at much lower intensities, around 0.25 TW/cm^2^, enabling efficient photocuring without the use of photoinitiators. These findings provide direct experimental validation that SZ2080^TM^ can undergo efficient photo-curing without added PIs, paving the way for more sustainable 3D printing practices by eliminating the use of potentially toxic polycyclic hydrocarbon-based additives. Furthermore, the determined nonlinear coefficients allow for precise estimation of the optimal conditions for efficient energy deposition, by accounting for the individual contributions of the pure resist, the photoinitiator, and their interaction. We believe that our experimentally determined material parameters can provide the first fundamental basis to interpret prior experimental results in laser-based polymerization [24], [25], [26].

## 2 Experimental

### 2.1 Z-scan measurements and formulae

**Figure 1:**
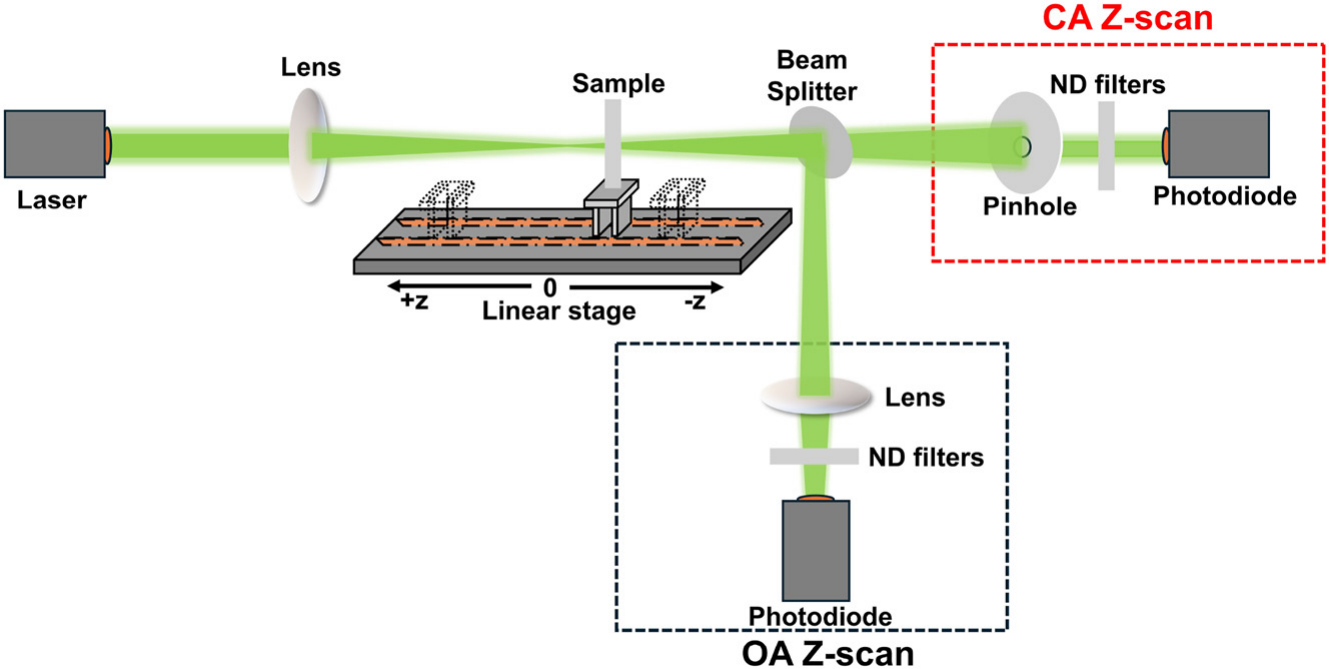
Schematic representation of the experimental setup simultaneously measuring closed and open aperture (CA, OA) Z-scans. Laser parameters: pulse duration = 240 fs, wavelength = 515 nm, repetition rate = 1 KHz.

The Z-scan is an established and widely used technique for investigating the NLO properties of a sample by measuring its normalized transmittance under varying laser radiation intensities [27]. A schematic representation of the Z-scan experimental setup is shown in Figure 1. In a typical Z-scan experiment, the sample is moved along the *z*-axis (the propagation direction of a focused laser beam) using a stepper motor, allowing it to experience different laser intensities at each position. The transmitted laser beam is split into two equal parts by a 50:50 non-polarising beam splitter, enabling simultaneous measurements of the sample’s transmittance in two distinct experimental configurations: “Open-aperture” (OA) and “closed-aperture” (CA) Z-scans. In the OA Z-scan configuration, the entire transmitted laser beam is collected by a lens and measured by a photodetector (in our case, a photodiode), offering insights into the sample’s NLO absorption. Simultaneously, in the CA Z-scan configuration, only the central part of the transmitted laser beam is measured after passing through a narrow pinhole positioned in the far field, just in front of a second photodetector. The latter configuration is sensitive to the beam’s phase front variations, thus providing insights into the sample’s NLO refraction. Therefore, the resulting OA and CA Z-scan recordings allow for the determination of the nonlinear absorption coefficient *β* and the nonlinear refractive index *n*_2_ of the sample, respectively.

The *β* and *n*_2_ values can be derived by fitting the experimentally obtained OA and CA Z-scans to Eqs. (1) and (2), respectively [15]:

(1) $$T_{OA}\left(z\right)=\frac{1}{\sqrt{\pi }\left(\frac{\beta I_{0}L_{\text{eff}}}{1+{\left(z/z_{0}\right)}^{2}}\right)}\overset{+\infty }{\underset{-\infty }{\int }}\ln\left(1+\frac{\beta I_{0}L_{\text{eff}}}{1+{\left(z/z_{0}\right)}^{2}}{\text{e}}^{-t}\right)dt,$$

(2) $$T_{CA}\left(z\right)=1-\frac{4n_{2}kI_{0}L_{\text{eff}}\frac{z}{z_{0}}}{\left[1+{\left(\frac{z}{z_{0}}\right)}^{2}\right]\times \left[9+{\left(\frac{z}{z_{0}}\right)}^{2}\right]},$$

where, *L*_eff_ is the sample’s effective length, *I*_0_ is the laser peak intensity, *z*_0_ is the Rayleigh length, and *k* is the excitation wavenumber. Then, from the determined *β* and *n*_2_ values, the imaginary Im*χ* ^(3)^ and real Re*χ* ^(3)^ parts of the third-order susceptibility *χ* ^(3)^ can be calculated using:

(3) $$Im\chi^{\left(3\right)}\;\left[esu\right]=10^{-7}\times \frac{c^{2}n_{0}^{2}\beta }{96\pi^{2}\omega },$$

(4) $$Re\chi^{\left(3\right)}\;\left[esu\right]=10^{-6}\times \frac{cn_{0}^{2}n_{2}}{480\pi^{2}},\;$$

where *c* is the speed of light, *n*_0_ is the refractive index and *ω* = 2*πν* ≡ 2*πc*/*λ* is the cyclic frequency of the laser pulse; *λ* is central laser wavelength. Since the esu-units are not SI, the dimensions of parameters are explicitly given here: *c* [m/s], *n*_0_ [none], *ω* [s^−1^], *β* [m/W], *n*_2_ [m^2^/W], Im*χ* ^(3)^ [m^2^/V^2^], Re*χ* ^(3)^ [m^2^/V^2^].

However, since *χ* ^(3)^ represents a macroscopic quantity that depends on the concentration of molecules in the sample, the second-order hyperpolarizability *γ* is often preferred. This parameter is a molecular constant that provides the NLO response at the level of individual molecules, thereby facilitating comparisons across different molecules. The value of *γ* can be calculated through:

(5) $$\gamma \;\left[esu\right]=\frac{\chi^{\left(3\right)}}{N\cdot L^{4}},$$

where *N* is the number of molecules/m^3^ and $L=\left(n_{0}^{2}+2\right)/3$ is the local field correction factor. Since the esu-units are not SI, the dimensions of parameters are explicitly given here: *N* [molecules/m^3^], *L* [none], *χ* ^(3)^ [m^2^/V^2^], *γ* [m^5^/V^2^].

Moreover, from the values of the nonlinear absorption coefficient *β*, the TPA cross-section *σ* can be deduced from:

(6) $$\sigma =\frac{h\nu \beta }{N_{A}\rho },$$

where *h* is the Plank’s constant, *v* is the frequency of light, *N*_*A*_ is the Avogadro number and *ρ* is the molar concentration. The *σ* is measured in the Goeppert–Mayer (GM) units (1 GM = 10^−50^ cm^4^ s photon^−1^). The esu-units of calculated entities are linked to the parameters in SI: *h* [J s], *v* [s^−1^], *N*_*A*_ = 6.022 × 10^23^ mol^−1^], *ρ* [M].

### 2.2 Samples and laser

The SZ2080^TM^ hybrid composite was synthesized using methacryloxy-propyl trimethoxysilane (MAPTMS, 97 %), zirconiumn-propoxide (ZPO, 70 % in propanol) and methacrylic acid (MAA, 99 %). The molar ratios to form the organic and inorganic network were MAPTMS:ZPO = 8:2 and ZPO:MAA = 1:1. Irgacure 369 (IRG369) and 4,4′-bis(diethylamino)benzophenone (BIS or Michler’s ketone) were utilized as PIs with a concentration 1 wt% to the final solution of MAPTMS and MAA composite. All materials were purchased from Sigma-Aldrich (Steinheim, Germany) and used in experiments without purification.

Two types of samples were prepared for the Z-scan experiments: one consisting of thin films on a 0.14 mm thick glass substrate, and the other a solution contained in a 1 mm glass cell. Thin films were fabricated on glass substrates to study the NLO properties of photosensitized and pure SZ2080^TM^. Films of pure SZ2080^TM^, and mixed with 1 wt% of IRG369 and BIS were prepared using the spin coating method at 5,000 rpm for 45 s. The thicknesses of the resulting films were measured by a profilometer (Mitutoyo, SJ-410) and found to be approximately 14 μm. To remove the impact of the possible non-uniformity of the films, the Z-scan measurements were done at multiple positions of the sample for each laser intensity. The collected data were then analyzed, and the average values of the NLO parameters were derived. This approach was applied consistently for all studied samples, ensuring that the obtained parameters were representative of the NLO properties of the films. The other type of samples was used to assess the contribution of neat BIS and IRG369 on the NLO properties of photosensitized SZ2080^TM^. The *z*-scan measurements were performed on 3 mM solutions of both compounds in dichloromethane (DCM) contained in 1 mm glass cells.

For the Z-scan experiments, an Yb-doped fiber laser (aeroPULSE FS10, NKT Photonics) was employed, delivering laser pulses with a pulse duration full width half maximum (FWHM) of 240 fs, a central wavelength at 515 nm (second-harmonic generation output), and a repetition rate of 1 kHz. Using a low repetition rate of the laser excludes the thermal accumulation effect on the nonlinear properties of the material.

Determining the TPA coefficient of SZ2080^TM^ with and without the photoinitiators of BIS and IRG369 at second harmonic radiation of Yb lasers (515 nm) is crucial because these compounds and lasers are used extensively in various applications, including industrial ones.

The use of the second harmonic also has the additional benefit of the increase of the pulse contrast, which for some of the lasers is important as they sometimes produce a nanosecond duration pedestal from the pumping diodes, which can contribute to the overall energy deposition, whilst in the process of second harmonic generation removes it. An additional benefit of the second harmonic use is the lowering effect of the free carrier absorption, which scales as *Iλ* ^2^ and can become important at the fundamental wavelength of near-IR wavelength, which is also popular in 3D printing; however, they require higher-order nonlinear absorption which provides less control of the polymerisation as the laser intensities that are needed to polymerise are close to the material breakdown threshold. In this regard, the determination of TPA and NLO refraction processes are separated from other contributing absorption mechanisms.

A quartz planoconvex lens with a focal length of 20 cm was used to focus the laser beam onto the samples. The beam radius, *w*_0_, at the focal plane was measured by a CCD camera and found to be approximately 18.0 ± 0.2 μm (at 1/*e* ^2^ maximum intensity), giving us a Rayleigh length $z_{0}=\pi \omega_{0}^{2}/\lambda$ of 2 mm in the air which is larger than the sample thickness. The Z-scan measurements were conducted over a wide range of incident laser intensities, ranging from 200 to 1,800 GW/cm^2^.

## 3 Results and discussion

### 3.1 Nonlinear optical properties and *β*

**Figure 2:**
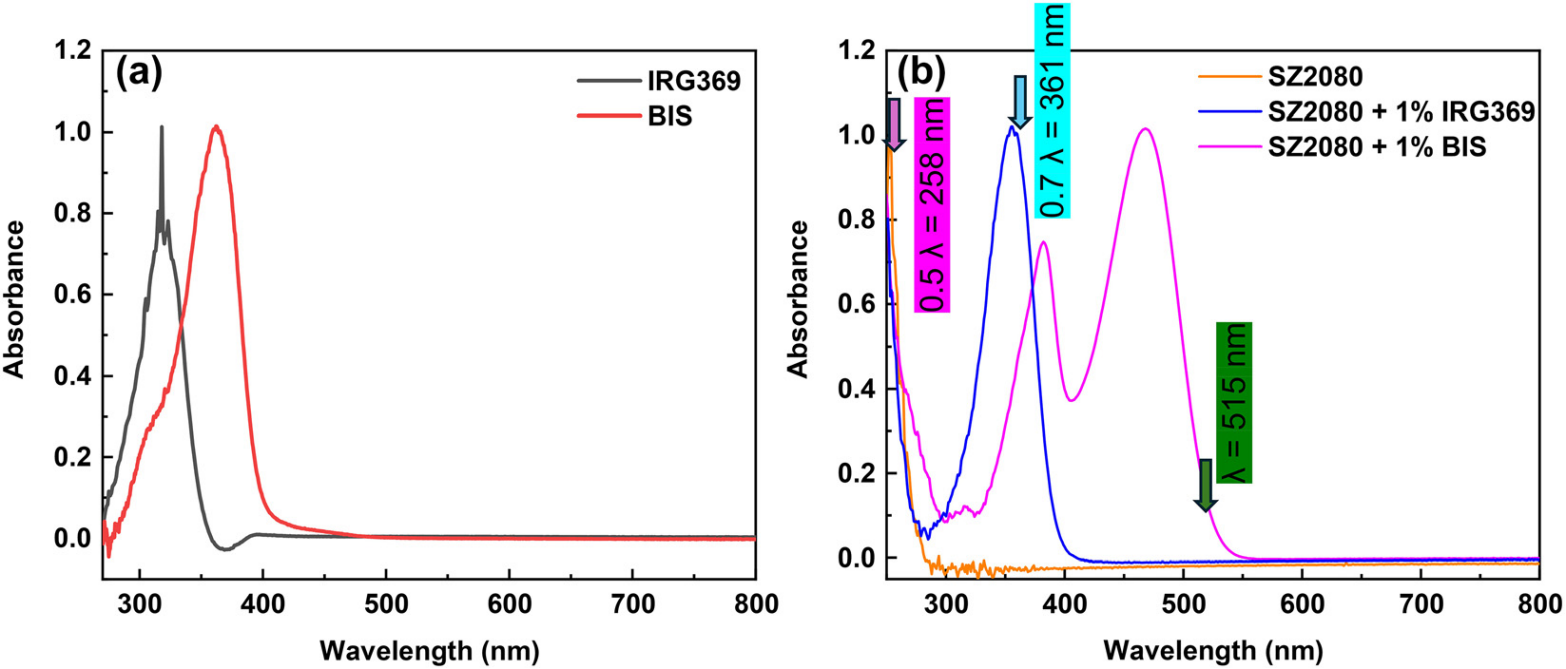
UV–vis absorption spectra of (a) IRG369 and BIS solutions in DCM, and (b) pure SZ2080^TM^ pre-polymer and SZ2080^TM^ pre-polymer films with photosensitizers. Note, the absorption peak of SZ2080^TM^ with IRG369 is centered at 350 ± 20 nm. The direct absorption band of pure SZ2080^TM^ is close to the 0.5*λ* of laser wavelength *λ* (marker in (b)). The 0.7*λ* spectral position of TPA maximum coincides with the IRG absorption band when it is mixed in pure SZ2080^TM^ resist. The thickness of solution was 1 mm and resist samples ∼14 μm. Note, very small negative *A* values are caused by variation of reference intensity *I*_0_ used for normalisation of measured transmitted intensity *I*_*T*_: *A* = − log_10_(*I*_*T*_/*I*_0_). Also, auto-fluorescence can be excited at short wavelengths and influence the absorbance measurements.

First, we analyzed the absorbance spectrum of each of the photosensitizers individually and in the presence of the pre-polymer. Figure 2(a) depicts the UV–vis absorption spectra of BIS and IRG369 3 mM solutions in DCM. Both compounds display a broad absorption band in the UV region, assigned to transitions from occupied *π* states to unoccupied π* states of the aromatic C=C bonds [28]. The absorption peaks are observed at 318 and 362 nm for IRG369 and BIS, respectively. Figure 2(b) presents the UV–vis absorption spectra of thin films, composed of pure and photosensitized SZ2080^TM^ pre-polymer. The results show that the addition of 1 % wt BIS to the SZ2080^TM^ pre-polymer resulted in the appearance of a new absorption band in the visible region, which was absent in the neat BIS compound. This new band likely arises from charge transfer interactions between electron-rich Michler’s ketone and the electron-deficient ZPO component of SZ2080^TM^. A similar behavior, due to the formation of a donor–acceptor complex, has been reported for *π*-conjugated systems mixed with fullerenes [29]. On the other hand, the addition of 1 % wt IRG369 to the SZ2080^TM^ pre-polymer did not cause the formation of new absorption bands, indicating the absence of similar charge transfer effects. It is worth noting that the absorption peaks of BIS and IRG369, which were originally at 362 and 318 nm, exhibited a red shift by 20 and 36 nm, respectively, after mixing with the SZ2080^TM^ pre-polymer. This shift may result from several factors, such as the sensitivity of Michler’s ketone and Irgacure series to variations in their dielectric environment [28], [30], *π* − π* interactions between aromatic rings of the PIs and the organic methacrylate group in SZ2080^TM^ as well as non-covalent interactions may occur between the amine groups of the PIs and the silanol groups (formed from the hydrolysis of trimethoxysilane) via hydrogen bonding. The arrows in the Figure 2(b) represent the central wavelength of the pump laser at *λ* = 515 nm, and fractional wavelengths 0.7*λ* = 361 and 0.5*λ* = 258 nm. These arrows show that for all the solutions, the single-photon absorption of the central pulse wavelength is minimal, and two-photon absorption is enough to excite not only present photosensitizers but also the pure pre-polymer.

In Figure 4(a)–(f), results of OA and CA Z-scan recordings of films of pure SZ2080^TM^, and SZ2080^TM^ mixed with 1 wt% IRG369 and BIS are depicted. The solid lines in each plot represent the best possible fit of the experimental data to Eqs. (1) and (2). Three distinct laser intensities were chosen to present observable variations in the OA and CA Z-scan data. The exception is for pure SZ2080^TM^ pre-polymer case when almost three times higher laser intensities were required to observe NLO absorption signal. The glass substrate showed negligible NLO absorption and refraction up to laser intensities of approximately 700 and 400 GW/cm^2^, respectively. Substrate contributions became detectable at higher laser intensities and were then subtracted from the total signal to isolate the NLO effects of SZ2080^TM^ pre-polymer. The measured OA Z-scan data (Figure 4(a)–(c)) clearly demonstrate that the samples’ transmittance exhibits a minimum at the focal point (i.e., *z* = 0), deepening as the incident laser pulse intensity increases. This shape of the OA Z-scan indicates the nonlinear absorption effect, i.e., a higher intensity pulse experiences higher absorption by the material. Given the existence of electronic states lying in the UV spectral region, as supported by the absorption spectra in Figure 2, the observed nonlinear absorption behavior can be explained in terms of TPA and/or multiphoton absorption (MPA) processes. Other mechanisms related to reversed saturable absorbers, such as excited state absorption and nonlinear scattering, can be ruled out, as these occur for longer laser pulses, typically in the ns regime [9].

**Figure 3:**
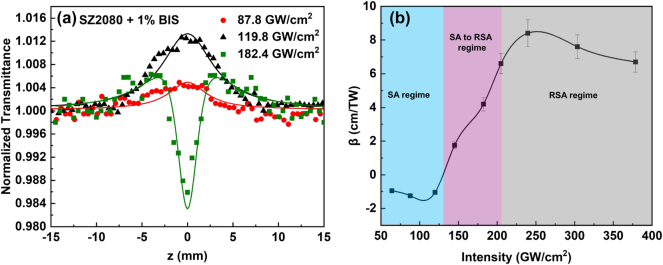
Nonlinear optical absorption properties of SZ2080^TM^ with BIS. (a) The OA Z-scans of SZ2080^TM^ pre-polymer with BIS under low laser excitation intensities. (b) Nonlinear absorption coefficient β of SZ2080^TM^ mixed with 1 % BIS against the peak intensity of excitation field. The shaded regions delineate the regimes of SA, RSA, and the transition between SA and RSA.

**Figure 4:**
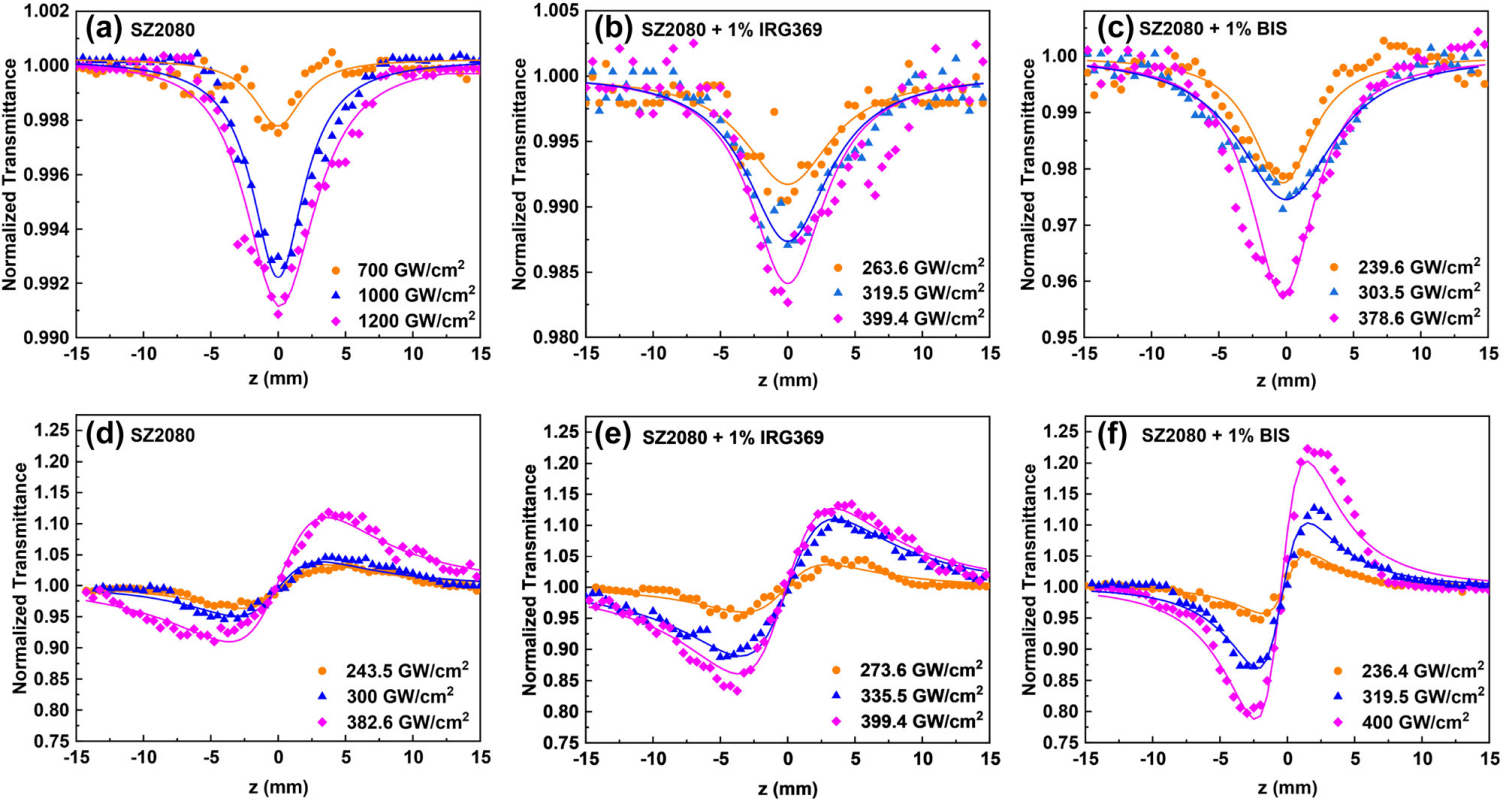
Z-scan recordings of SZ2080^TM^ with and without photosensitizer. (a–c) OA and (d–f) CA Z-scans of pure SZ2080^TM^ pre-polymer and SZ2080^TM^ pre-polymer with photosensitizer, under different laser excitation intensities.

As shown in Figure 3(a), the TPA/MPA process in BIS-doped SZ2080^TM^ film was manifested using incident laser radiation exceeding ∼130 GW/cm^2^. At lower pulse intensities, single-photon absorption (1PA) also contributes to the NLO absorptive response, resulting in saturable absorption effect. This is attributed to the linear absorption at the laser excitation wavelength of 515 nm, resulting from a low population density state. The OA Z-scan data of Figure 3, corresponding to SA, can be well fitted with Eq. (1), yielding a negative nonlinear absorption coefficient for 1PA of *β* = (−11.5 ± 1.0) × 10^−1^ cm/TW. With increasing laser intensity, a dip appeared at the center of the transmission maximum, denoting a transition from SA to RSA, where 1PA and 2PA coexist. In this scenario, when SA and RSA interplay, the normalized transmittance is described by the following relation [31]:

(7) $$T\left(z\right)=\overset{\infty }{\underset{m=0}{\sum }}\frac{{\left(\frac{-\alpha I_{0}L_{\text{eff}}}{1+{\left(z/z_{0}\right)}^{2}}\right)}^{m}}{m+1},$$

where

(8) $$\alpha =\frac{\alpha_{0}}{1+I/I_{s}}+\beta I$$

represents the modified intensity-dependent absorption coefficient. In these equations, *α*_0_ is the linear absorption coefficient, *β* is the two-photon absorption coefficient, *I*_*s*_ is the saturation intensity, *L*_eff_ is the sample’s effective length, *z*_0_ is the Rayleigh length, and *m* is the summation index in an infinite series. Fitting the measured SA-to-RSA transition recordings with Eq. (7), the intensity dependence of the *β* values in the SA to RSA regime were determined and is presented in Figure 3(b). From the same Eq. (7), the saturation intensity *I*_*s*_, defined as the laser intensity at which SA begins to saturate and RSA starts to contribute significantly, was determined to be 130 ± 7 GW/cm^2^. At sufficiently high laser intensities, where 2PA become more efficient, a clear RSA behavior was observed, as depicted in Figure 4(c). Fitting the experimental data in Figure 4(c) with Eq. (1) yielded a positive *β* value of 6.7 ± 0.6 cm/TW (at intensity ∼0.38 TW/cm^2^).

The measured CA Z-scans representative of the NLO refraction of the samples is depicted in Figure 4(d)–(f). The experimental data show a valley-peak configuration, indicating a self-focusing behavior corresponding to a positive nonlinear refractive index *n*_2_. This response is driven by the instantaneous electronic cloud distortion (electronic response), which occurs due to a strong ultrashort laser electric field. It should be highlighted that the contribution of thermal lensing to the observed NLO refraction can be regarded negligible in our measurements, as thermal lensing typically manifests as self-defocusing, in the long run [32], an effect that was not observed in the recorded data. The shift of the sample to a fresh location does not change the recorded value, and no change in signal dependence on time was observed, indicating no observable heat accumulation effect. To quantify and distinguish the contributions of pure SZ2080^TM^ and photosensitizers to energy deposition, the optical nonlinearities of neat IRG369 and BIS were separately investigated, performing Z-scan experiments under similar excitation conditions. The obtained Z-scans are depicted in Figure A2, where, as can be seen, both photosensitizers exhibit TPA and self-focusing similar to SZ2080^TM^.

**Figure 5:**
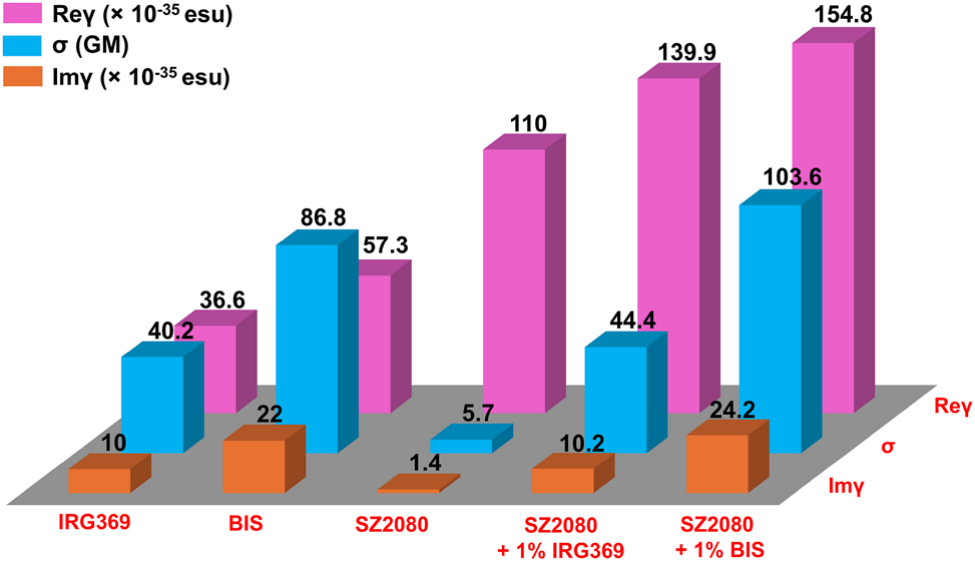
The magnitude of the real Re(*γ*) and imaginary Im(*γ*) part of the second-order hyperpolarizability and two-photon absorption cross-section *σ* of pure photosensitizers (IRG369 and BIS), pre-polymer SZ2080^TM^, and SZ2080^TM^ pre-polymer with photosensitizers.

By fitting the OA and CA Z-scans of Figure 4 using Eqs. (1)–(6), the TPA absorption coefficient *β*, the nonlinear refractive index *n*_2_, the imaginary and real parts of third-order susceptibility (Im*χ* ^(3)^, Re*χ* ^(3)^) and second-order hyperpolarizability (Im*γ*, Re*γ*), along with the two-photon absorption cross sections *σ*, were calculated for pure SZ2080^TM^, photosensitized SZ2080^TM^, and neat photosensitizers. The results are gathered in Table A1. It is noteworthy that among these NLO related parameters, only Im*γ*, Re*γ* and *σ* provide a figure of merit for comparing the NLO properties of pure and photosensitized SZ2080^TM^, as these are molecular constants independent of the molecular density. These parameters are schematically represented in Figure 5 for comparison purposes. As evidenced, the pure SZ2080^TM^ pre-polymer exhibits inherent NLO absorption properties, indicating its capability to initiate photopolymerisation without using a PI. However, its NLO absorption is substantially weaker than the photosensitized SZ2080^TM^. Adding 1 % wt of BIS and IRG369 increased Im*γ* and *σ* coefficients by approximately 7.5-fold and 17-fold, respectively.

In the SZ2080^TM^/BIS mixture, Michler’s ketone (the chromophore in BIS) is electron-rich due to its two dimethylamino substituents, while zirconium propoxide (ZPO), a component of SZ2080^TM^, is electron-deficient owing to the strong Lewis acidity of its Zr^4+^ centers. This electron-rich/electron-deficient pairing facilitates intermolecular charge transfer, potentially contributing to the observed enhancement in the determined two-photon absorption coefficients compared to pure SZ2080^TM^. Similar charge transfer-induced enhancements in nonlinear absorption have been reported for a variety of push-pull molecular systems, as seen in references [33], [34], [35], [36]. However, as discussed above, the addition of 1 % wt IRG369 to the SZ2080^TM^ pre-polymer does not induce comparable charge transfer processes, which explains the significantly lower enhancement in nonlinear absorption observed when IRG369 is mixed with the SZ2080^TM^ pre-polymer. Additionally, the confinement of IRG369 and BIS compounds within the SZ2080^TM^ matrix, leads to the formation of *π* − π* interactions between the aromatic rings of the PIs and the organic methacrylate group in SZ2080^TM^ as well as the generation of hydrogen bonds between the amine groups of the PIs and the silanol groups formed from the hydrolysis of trimethoxysilane. These non-covalent interactions restrict the rotational and vibrational freedom of PIs, thereby enhancing their electronic coupling. As a result, the transition dipole moment associated with TPA in the SZ2080^TM^/PI mixtures increases (*σ* scales with the fourth power of the transition dipole moment). Furthermore, because the PIs are embedded in the polymer matrix, their nonlinear response may be further enhanced due to local field effects arising from interactions with the dielectric environment. Such local field enhancements have been shown to improve the efficiency of multi-photon polymerization [37].

**Figure 6:**
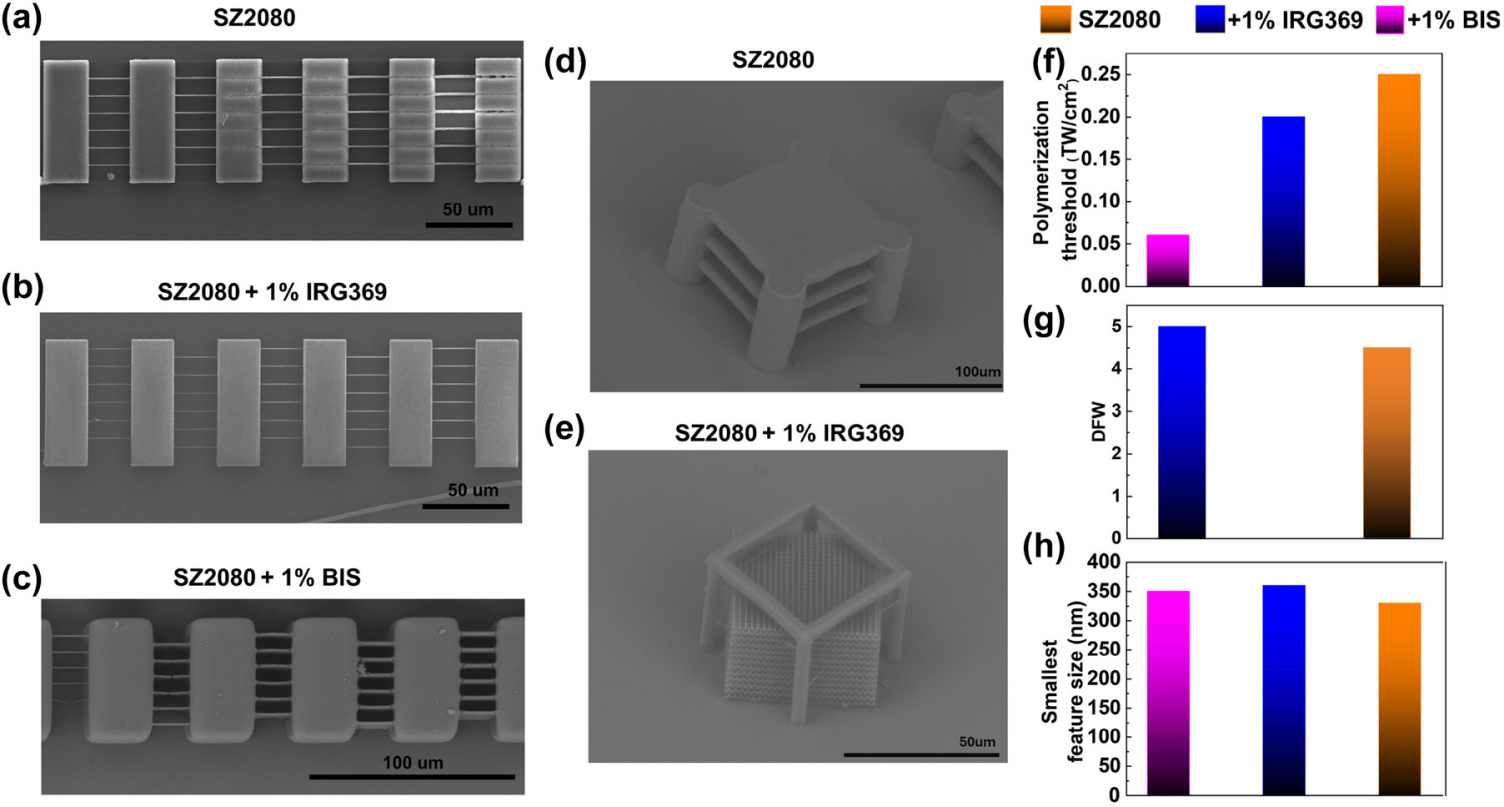
SEM images and quantitative analysis of polymerized 3D structures of SZ2080^TM^ with and without PI. 3D bridges for the detection of polymerization threshold and structural integrity of (a) pure SZ2080^TM^, (b) SZ2080^TM^ mixed with 1 % IRG369, and (c) SZ2080^TM^ mixed with 1 % BIS. Complex 3D geometries fabricated at optimized exposure conditions are shown for (d) pure SZ2080^TM^ and (e) SZ2080^TM^ mixed with 1 % IRG369. Laser exposure parameters: 515 nm wavelength, 240 fs laser pulse, 0.1 MHz repetition rate, scanning speed of 100 μm/s, and numerical aperture NA = 1.4. (f) Measured polymerization thresholds, (g) dynamic fabrication windows (DFW), and (h) smallest achieved feature sizes for each compound. It is noteworthy that due to the high-repetition ( >0.1 MHz) rate, a strong pulse-to-pulse overlap between adjacent spots contributes to additional thermal and accumulation effects affecting the final polymerization [17]; however, these are beyond the scope of this study, which focuses instead on isolated two-photon absorption (*β*) and nonlinear refractive index (*n*_2_) effects responsible for nonlinear energy deposition by single pulses.

The SEM images and quantitative analyses shown in Figure 6 demonstrate nicely that SZ2080^TM^ can undergo photocuring even in presence of a PI, while highlighting the impact of PI additives on the polymerization behavior and structural fidelity of SZ2080^TM^-based resists. To determine the polymerization threshold and dynamic fabrication window (DFW), a bridge-structure method was employed. This approach involved the periodic increase of laser intensity during the fabrication of line arrays, from the onset of polymerization to the point of visible overexposure. Representative bridge structures for pure and photosensitized SZ2080^TM^ are shown in Figure 6(a)–(c). These structures were fabricated over a controlled range of laser intensities, starting from the minimum dose required to generate continuous single-scan lines (i.e., the polymerization threshold) and extending to the threshold at which overexposure effects became obvious. Statistical analysis of several bridge structures, fabricated across different batch of samples, was used to accurately extract the values of polymerization threshold and DFW. These results are gathered in Figure 6(f) and (g), showing a reduced fabrication threshold and a broadened DFW in photosensitized SZ2080^TM^ compared to pure SZ2080^TM^, in accordance with previous observations [16], [17], [25]. This behavior is explained by the enhanced nonlinear absorption properties of photosensitized SZ2080^TM^, arising from the interaction between the pre-polymer matrix and the PIs, as discussed in detail previously. It is noteworthy that for the case of SZ2080^TM^ mixed with BIS, the corresponding DFW was not able to be determined, since it showed compromised structural fidelity increasing the laser power, probably attributed to cumulative thermal effects facilitated by the high repetition rate of 0.1 MHz and the low linear absorption at the excitation wavelength.

Between the two photosensitized SZ2080^TM^ pre-polymers examined the BIS-containing sample demonstrated stronger TPA, resulting in a lower fabrication threshold than the IRG369-containing sample. These improved properties can be ascribed to increased *π*-electron delocalization, due to the formation of an efficient charge transfer system, evidenced by the red-shifted absorption spectrum of BIS doped SZ2080^TM^, likely due to the greater aromaticity of BIS compared IRG369 and the charge transfer interactions. The correlation between large nonlinear absorption coefficients, low polymerization thresholds, and wide DFW has been demonstrated for dozen of PIs [38], [39]. However, the relationship between nonlinear response to the DFW remains debated [40], as other factors, such as the material’s damage threshold, and thermal diffusivity can affect it. As we have shown, such studies should also account for the contribution of the polymer matrix, which has a considerable contribution to energy deposition and can significantly affect the polymerization threshold and DFW.

### 3.2 Nonlinear refraction *n*_2_

It should be emphasized that NLO refraction, specifically self-focusing, is expected to influence the laser beam propagation through the material, therefore, the deposited laser energy distribution, i.e., the voxel shape and, consequently, the resolution in multi-photon lithography (MPL). Thus, from the determined Re*γ* values, valuable insight into the impact of NLO refraction on the feature size of microstructures fabricated via MPL can be estimated. To the best of our knowledge, this is the first study, establishing a correlation between NLO refraction and resolution in MPL, particularly using the widely adopted SZ2080^TM^ pre-polymer.

Results depicted in Figure 5 demonstrate that the NLO refraction coefficient (Re*γ*) of the pure SZ2080^TM^ pre-polymer is significantly stronger than its corresponding NLO absorption (Im*γ*). However, despite the addition of the photosensitizers, the value of the Re*γ* does not change significantly. The biggest change was observed when the BIS photosensitizer was added, and it changed only by ∼40 %. On the contrary, the NLO absorption has increased by 20 times. Only a small variation in the NLO refraction coefficient allows us to obtain consistent feature sizes measured in the microstructures fabricated with and without using a PI. Furthermore, the detailed estimation of the effect of NLO refraction on nonlinear propagation is presented in Supplementary A, showing that the refractive index *n* change of pure and photosensitized SZ2080^TM^ remains small under laser radiation used for photopolymerization (i.e., *n*_2_ *I* ≪ *n*_0_), indicating that high resolution is achievable due to the precise energy deposition. Due to the small variation in the refractive index *n*, the feature size of 3D structures remains practically constant regardless of the mixture of SZ2080^TM^ pre-polymer with the photosensitizer, as shown in Figures 6(a)–(c) and A3. This small variation in *n* explains the consistency observed in previous studies [16], [17]

Apparently, the NLO refraction is dominated by the polymer host (Table A1) rather than the photosensitizer, opposite to its effect on nonlinear absorption. The NLO refraction and nonlinear absorption can act as a positive feedback system: self-focusing contributes to the beam shrinkage, while the nonlinear absorption lowers the photopolymerization and damage of the material threshold, i.e., both effects lower the intensity needed for laser polymerization. It is noteworthy that the highest resolution 3D polymerization is carried out at very tight focusing with objective lenses with numerical apertures NA = 0.7 − 1.4. Therefore the propagation of the beam is limited over a very short length, and self-focusing is significantly suppressed as the accumulation of the phase change is confined within the very short focal region. To further substantiate the conclusion that self-focusing is negligible under tight focusing conditions, we note that the calculated nonlinear propagation lengths (*L*_*n*_) for pure SZ2080^TM^ and for SZ2080^TM^ mixed with BIS and IRG369 (provided in the Supplementary Information A) are, in all cases, significantly longer than the corresponding Rayleigh lengths associated with the high-NA focusing in 3D polymerization. Therefore, self-focusing does not significantly influence the feature size or resolution of the fabricated microstructures.

All the experiments for determining optical nonlinearities were carried out at approximately twice lower peak intensities than those required for the substantial free carrier generation that leads to the material breakdown. We have observed a clear sign reversal of *n*_2_ ≈ −5.6 × 10^−4^ cm^2^/TW in CA Z-scan at intensity of 1.8 TW/cm^2^ for pure SZ2080^TM^ (Figure A1; see discussion in Supplementary A). In our experimental conditions (low repetition rate, ultrashort pulses), the Z-scan can measure only ultrafast (instantaneous) material responses contributed only by the electronic interactions with the laser radiation. Therefore, the reversal of the *n*_2_ sign is attributed to the contribution of the generation of free carriers and their de-focusing effect, which depends on their density. Consequently, the defocusing effect at a specific free carrier density can overcome the self-focusing Kerr effect. Increasing the laser intensity above the 1.8 TW/cm^2^ value will lead to material breakdown, i.e., material damage. At the dielectric breakdown, the real part of permittivity reduces to zero (*ɛ*′ = *n* ^2^ − *κ* ^2^ ≡ 0) due to free carrier generation and plasma formation (with the imaginary part *ɛ″* = 2*nκ*). This will lead to a complex refractive index $\tilde{n}=\sqrt{\varepsilon^{\prime }+i\varepsilon^{\prime \prime }}=n+i\kappa$ having only imaginary part and thus the material being highly reflective with the large absorption coefficient *α* = 4*πκ*/*λ*.

Concerning the timescale of the proposed free carrier contribution, we recognize that a precise determination of free carrier dynamics would require time-resolved experiments, such as pump-probe spectroscopy, which lie beyond the scope of the present study. However, based on well-established literature for similar dielectric materials, free carrier generation via multiphoton ionization typically occurs on femtosecond timescales, nearly simultaneously with the laser pulse [41]. Avalanche ionization follows more slowly, building up free carriers over picoseconds through electron impact processes [41]. These free carriers contribute to a negative nonlinear refractive index, overwhelming the positive Kerr effect and resulting in the observed transition from self-focusing to self-defocusing at high intensities.

Free-carrier induced nonlinearities are commonly associated with self-defocusing in various materials [42], [43], and can be effectively identified and characterized by the Z-scan technique [42]. Furthermore, theoretical modeling based on the nonlinear Schrödinger equation supports this interpretation [44]. Particularly, simulations incorporating Kerr self-focusing, plasma defocusing, multiphoton ionization, and avalanche ionization mechanisms have shown that for Fourier-transform-limited pulses (i.e., femtosecond pulses), a decrease in on-axis energy deposition occurs due to free carrier induced defocusing effects. In contrast, for longer pulse durations (e.g., a few picoseconds), the plasma builds up later during the pulse, allowing more energy to remain confined on-axis and leading to a steep increase in deposited energy.

## 4 Conclusions and outlook

Optical nonlinearities governing the energy deposition in 3D polymerisation by laser direct writing with 515 nm/240 fs pulses were directly measured using Z-scan reaching intensities up to 1.8 TW/cm^2^ – just below the material breakdown threshold – in SZ2080^TM^ resist, both with or without added photoinitiator.

Our results showed that the deposited dose per pulse has cumulative contributions from both the PI (with volume or weight fraction *f* ≈ 1 %) and from the host polymer (with fraction 1 − *f* ≈ 99 %). Although the measured nonlinear coefficients of PI were nearly 10 times larger than the host polymer, the latter’s substantially larger volume fraction (almost 100 times) allows it to dominate the nonlinear contributions. Thus, despite the measured TPA of the SZ2080^TM^ being much lower than PI, polymerization can effectively initiated without the presence of PI, confirming recent studies [24]. Interestingly, the measured TPA of the host polymer-PI mixture was even higher than the PI alone, which suggests a strong interaction of the PI and the host material. This interaction facilitates the absorption of higher levels of laser pulse energy, which is expected to lower the onset of photo-polymerization. Moreover, a very small linear absorption contribution in the case of a mixture with BIS PI is determined (Figure 2). It showed saturable absorber trends in the Z-scans at low intensities but was quickly overwhelmed by the TPA at an increased intensity of the pulse. Similar trends were also observed for the nonlinear refraction coefficients, i.e., the mixture had a higher nonlinear refractive index than the PI.

The presented determination of *β* and *n*_2_ for pure materials and their mixture allows for an exact modeling of energy deposition in laser-based 3D printing. Most importantly, the determined coefficients for pure SZ2080^TM^ provide experimental evidence that it can initiate polymerization without the need of any PI, promoting a “greener” and environmentally safer manufacturing approach by eliminating the use of toxic additives usually found in PIs.

## Supplementary Material

Supplementary Material Details
