# Supplementary material for: Direct measurement of two-photon absorption and refraction properties of SZ2080TM-based resists at 515 nm: insights into 3D printing
Source: Nanophotonics. 2025 Aug 5;14(18):2981–92. doi: 10.1515/nanoph-2025-0066 (PMC12442362; doi:10.1515/nanoph-2025-0066)
Supplement: Supplementary file 1 — Supplementary Material Details [file j_nanoph-2025-0066_suppl_001.pdf]

Compound	N_0 10^{22} molecules/m^3	β 10^{-15} m/W	n_2 10^{-21} m^2/W	$Im\chi^{(3)}$ 10^{-16} esu	$Re\chi^{(3)}$ 10^{-16} esu	$Im\gamma$ 10^{-35} esu	$Re\gamma$ 10^{-35} esu	σ GM
Glass	–	5.3 ± 0.2	20.6 ± 0.7	1.92 ± 0.07	29.4 ± 1.0	–	–	–
DCM	–	21.2 ± 1.0	21.6 ± 1.0	6.9 ± 0.3	27.3 ± 1.0	–	–	–
SZ2080™	270.6	4.0 ± 0.3	85.3 ± 6.0	1.5 ± 0.1	121.7 ± 9.0	1.4 ± 0.1	110 ± 13.0	5.7 ± 0.4
IRG369	164.7	17.2 ± 2.0	15.6 ± 1.0	5.5 ± 0.7	20.1 ± 1.0	10.0 ± 0.6	36.6 ± 2.0	40.2 ± 5.0
SZ2080™+IRG369	271.79	31.3 ± 3.0	108.5 ± 4.0	11.3 ± 0.8	154.8 ± 6.0	10.2 ± 0.7	139.9 ± 5.0	44.4 ± 5.0
BIS	185.4	41.7 ± 2.0	27.5 ± 2.0	13.6 ± 0.7	35.4 ± 3.0	22.0 ± 1.0	57.3 ± 5.0	86.8 ± 4.0
SZ2080™+BIS	271.94	73.0 ± 6.0	133.3 ± 8.0	26.4 ± 2.0	171.4 ± 10.0	24.2 ± 2.0	154.8 ± 9.0	103.6 ± 14.0

Table A1. Experimentally determined values using Z-scan (this study). NLO related parameters (β: nonlinear absorption coefficient, n_2: nonlinear refractive index, $Im\chi^{(3)}$: imaginary part of the third-order susceptibility, $Re\chi^{(3)}$: real part of the third-order susceptibility $Im\gamma$: imaginary part of second-order hyperpolarizability, $Re\gamma$: real part of second-order hyperpolarizability, and σ: two-photon absorption cross section [1 GM = 10^{-50} cm^4·s·photon^{-1}] of pure SZ2080™pre-polymer, and SZ2080™pre-polymer with photosensitizers, and neat photosensitizers (IRG369 and BIS), under 240 fs, 515 nm laser excitation. The parameters are given in standard SI and cgs/esu units.

A. Supplementary Information

Table A1 shows the summary of the experimentally determined (Z-scan) parameters in the most popular presentations using formulae Eqns. 1–6. All data are obtained in this study using the same setup and sample preparation protocols.

Figure A1 shows CA Z-scans of SZ2080™pre-polymer at higher intensity 1.8 TW/cm^2. The clear sign reversal of n_2 is observed. It is likely attributed to the onset of free carrier generation. As the free carrier density increases due to ionization, the refractive index decreases because ω_p^2 becomes larger, resulting in self-defocusing. More detailed studies are planned to explore the mechanism that changes material response from solid state to plasma using Z-scan at different PI doping.

The refractive index of plasma from the Drude model is given by $n = \sqrt{1 - \omega_p^2/\omega^2}$, where $\omega_p = \sqrt{\frac{N_e e^2}{\epsilon_0 m_e}}$ is the plasma frequency defined by the electron density N_e, where m_e is electron mass, e its charge and $\omega = 2\pi c/\lambda$ is the cyclic laser frequency, ϵ_0 is the dielectric constant (permittivity of free space). As electron density is increasing and approaching the critical plasma density N_{cr}: $\frac{\omega_p^2}{\omega^2} = \frac{N_e}{N_{cr}}$, light is reflected.

Figure A2 has summary results of OA and CA Z-scan of PI solutions and DCM solvent (1 mm thickness cuvette, 36 μm diameter irradiation spots). The results confirm self-focusing with $n_2 > 0$.

Figure A3 presents the smallest achievable lateral feature size of line arrays fabricated using pure and photosensitized SZ2080™ resist. All formulations demonstrate sub-micron feature sizes ranging from 330–350 nm.

Case study of Two Photon Polymerisation (TPP). Lets estimate the first nonlinear (I-dependent) contribution to the refractive index $n = n_0 + n_2 I$ and absorption coefficient $\alpha = \alpha_0 + \beta I$ for the pure SZ2080™and with 1% wt. IRG369 and BIS at the typical 3D polymerisation intensity (average) $I =$ 1 TW/cm^2. For the pure SZ2080™resist, the linear index is $n_0 = 1.50 \pm 0.02$ at visible wavelengths [45] and the absorption coefficient $\alpha_0 \approx 1$ cm^{-1} at the transparency window (14 μm-thick resist ; Fig. 2(b)). The threshold of strong absorption is defined by optical density $\alpha d = 1$, hence, the transmittance $T = I_T/I_0 = e^{-\alpha d} \equiv 1/e$, d is the thickness at which intensity of the beam reduces by e for linear absorption case. Notably, the scattering losses can be assumed to be due to absorption when measured in transmission. Two-photon nonlinearites of SZ2080™resist: $\beta \approx 0.4$ cm/TW and $n_2 \approx 9 \times 10^{-4}$ cm^2/TW (Table A1; units are changed to cm/TW and cm^2/TW for easy estimates at intensities in TW/cm^2). At 1 TW/cm^2 the nonlinear additions to α_0 and n_0: $\beta I = 0.4$ cm^{-1} and $n_2 I = 9 \times 10^{-4}$, respectively. Hence, the absorption length $d = 1/\beta I = 25mm$, the nonlinear length is calculated by: $L_n = \lambda/n_2 I = 0.57$ mm. The nonlinear length is the distance at which the phase will differ by 2π due to nonlinear refraction. Therefore, for the nonlinear refraction to have an impact, the wave has to travel 0.57 mm, which is a considerable distance compared to the Rayleigh length of a tightly focused beam usually used in 3D photopolymerisation ($NA > 0.7$). In laser-based 3D polymerization, the Rayleigh length typically ranges from $\simeq$ 1–2 µm for $NA = 0.7$ to $\simeq$ 300–400 nm for $NA = 1.4$, assuming wavelengths around 500–800 nm. Meanwhile, a small amount of absorbed energy is sufficient to induce photopolymerization. This favors the energy deposition with high resolution and precision when high NA is used.

For SZ2080™with 1%wt. IRG369, at 0.4 TW/cm^2, the absorption depth d=8 mm; and $L_n = 0.23$ mm. Correspondingly, for SZ2080™with 1%wt. BIS, at 0.4 TW/cm^2 : d=3.4 mm and $L_n = 0.19$ mm. Again, distances are larger than the Rayleigh length of the tightly focused beam.

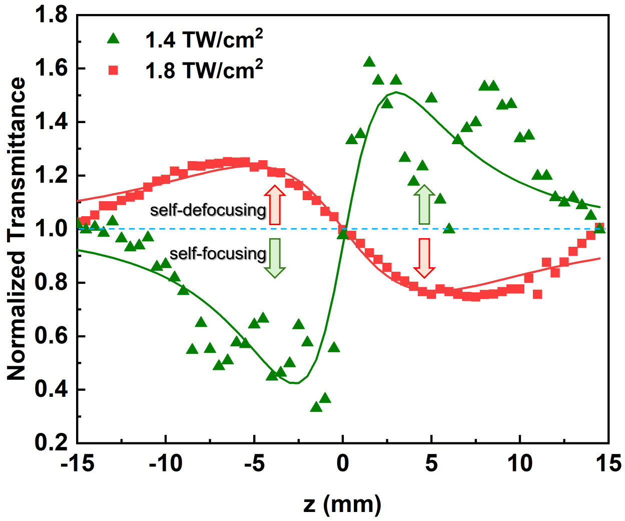

Fig. A1. CA Z-scans of SZ2080™ pre-polymer at a laser intensity of 1.4 TW/cm^2 (green triangles) and 1.8 TW/cm^2 (red squares). The sign reversal of $n_2 \approx -5.6\times10^{-4}$ cm^2/TW was observed. This indicates self-defocusing due to free carriers. As the free carrier density increases due to ionization, the refractive index decreases because plasma frequency $\omega_p = \sqrt{\frac{N_e e^2}{\epsilon_0 m_e}}$ becomes larger, resulting in self-defocusing effect. Larger S/N ratio for self-defocusing is observed.

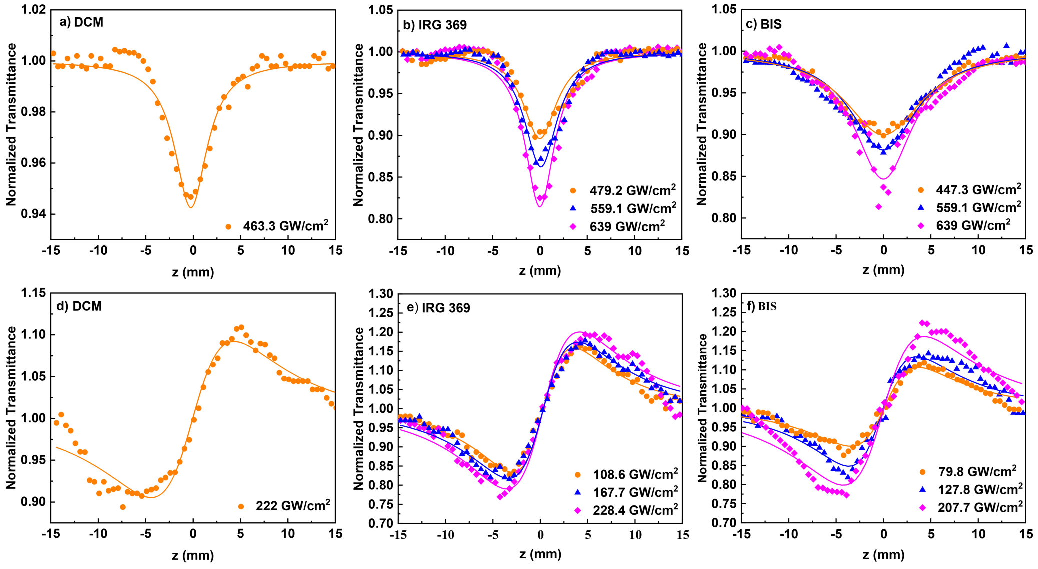

Fig. A2. (a-c) OA and (d-f) CA Z-scans of IRG369 and BIS solutions in DCM, under different laser excitation intensities.

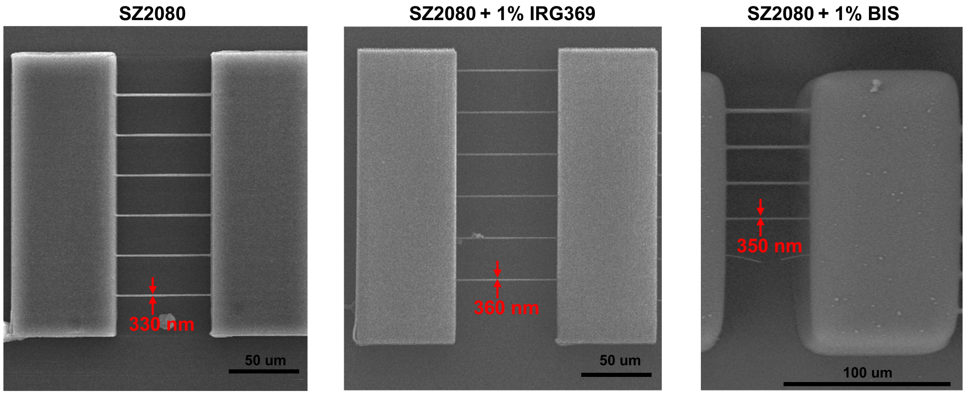

Fig. A3. SEM images of fabricated line arrays used for resolution characterization of SZ2080™-based resists with and without photosensitizer. The minimum lateral feature size, defined as the line width at threshold conditions, is indicated for each material: (left) neat SZ2080™, (middle) SZ2080™+ 1% wt. IRG369, and (right) SZ2080™+ 1% wt. BIS. Notably, all formulations attain sub-micron feature sizes ranging from 330–350 nm. Scale bars: 50 µm (left and middle), 100 µm (right).
